# Determinants of Health and Performance in Wildland Firefighters: A Narrative Review

**DOI:** 10.3390/jfmk10010080

**Published:** 2025-02-27

**Authors:** Fabio García-Heras, Jorge Gutiérrez-Arroyo, Juan Rodríguez-Medina, Belén Carballo-Leyenda, Pilar Sánchez-Collado, Gerardo Villa-Vicente, Jose A. Rodríguez-Marroyo

**Affiliations:** VALFIS Research Group, Department of Physical Education and Sports, Institute of Biomedicine (IBIOMED), University of Leon, 24071 León, Spain; jgutia@unileon.es (J.G.-A.); jrodm@unileon.es (J.R.-M.); abcarl@unileon.es (B.C.-L.); mpsanc@unileon.es (P.S.-C.); jgvilv@unileon.es (G.V.-V.)

**Keywords:** occupational health, forest firefighting, physiological demands

## Abstract

Background/Objectives: Wildland firefighters (WFFs) are subjected to significant physical and physiological demands that expose them to substantial occupational risks, including thermal stress, prolonged physical exertion, and exposure to harmful substances. These factors not only affect their immediate performance but also have long-term implications for their health. This narrative review seeks to analyze the main factors influencing the health and performance of WFFs, with a particular focus on physical, environmental, and psychological challenges. Methods: A narrative review was performed, synthesizing data from diverse sources. The analysis centered on studies addressing the physiological, environmental, and psychological aspects of WFF performance. Specific topics included physical workload, exposure to environmental stressors, use of protective equipment, hydration, sleep patterns, and mental health. Results: The review highlights several critical challenges faced by WFFs, including the extreme physical demands of carrying heavy equipment during extended interventions, elevated physiological strain induced by protective gear, and significant health risks associated with smoke inhalation and dehydration. Additionally, inadequate sleep and heightened mental stress were found to impair both cognitive and physical performance. Variations in injury prevalence and patterns of chronic pain were observed, often influenced by factors such as sex, age, and professional experience. Conclusion: To mitigate these risks and enhance the health and performance of WFFs, targeted interventions are essential. These include tailored physical training programs, heat acclimatization strategies, and improved resource management. Future research should aim to integrate these measures comprehensively and address existing knowledge gaps to ensure the long-term well-being of these professionals.

## 1. Introduction

Wildland firefighters (WFFs) face unique physical and physiological challenges distinct from those encountered by other first responders. Their role involves prolonged exposure to extreme environmental conditions, including high temperatures, smoke inhalation, and uneven terrain, all while carrying heavy loads during extended interventions [1,2,3]. These demands place significant strain on their physical performance and long-term health. Unlike structural firefighters or police officers, WFFs often operate in remote locations with limited access to resources, increasing their vulnerability to dehydration, heat strain, and injuries [4,5,6]. Addressing these specific occupational risks requires a tailored approach that prioritizes physical fitness, mental resilience, and targeted interventions to mitigate the cumulative effects of their work environment [6,7].

Recent studies have identified fatigue as a critical issue for first responders, particularly those engaged in full-time duties, highlighting the importance of maintaining optimal physical fitness to meet the operational demands that directly affect their performance and health [8]. Given that physical performance naturally declines with age, regular physical fitness assessments are recommended to ensure that first responders can safely and efficiently perform their duties [6,7].

This review focuses on the key factors influencing the health and performance of WFFs, with occasional references to structural firefighters, given the shared characteristics of their roles. The scope encompasses various challenges, including emergency response, terrain conditions, smoke exposure, and their cumulative impact on health. Moreover, thermal and environmental stressors, the intensity and duration of physical effort, hydration, rest, use of personal protective equipment, and physical workload play a significant role in shaping performance outcomes. Mental health challenges, injury prevalence, and chronic pain are also critical determinants in this field. This narrative review delves into each of these factors to provide a comprehensive understanding of their effects on the health and performance of WFFs, with the aim of identifying strategies to mitigate risks and enhance their well-being.

## 2. Methods

This narrative review was conducted using a comprehensive keyword-based search strategy across four major databases: PubMed, Scopus, Web of Science, and Google Scholar. The search terms included combinations of “wildland firefighters”, “firefighters”, or “first responders” with “health”, “performance”, and additional terms such as “emergency”, “terrain conditions”, “personal protective equipment”, “dehydration”, “thermal stress”, “smoke exposure”, “energy expenditure”, “mental health”, and “injury”.

We included peer-reviewed international articles published in English, encompassing reviews, original research, online reports, and e-books. A snowball sampling method was also employed by examining the references cited in the identified studies to capture additional relevant literature [9]. Each source was independently evaluated by a member of the research team to determine its inclusion. The criteria focused on studies addressing determinants of health and performance in WFFs, such as physical workload, environmental stressors, mental health challenges, and their impacts on occupational outcomes.

Although this review is not exhaustive, it synthesizes key findings from the literature to provide a comprehensive overview of the health risks and performance challenges faced by wildland firefighters. No temporal restrictions were applied to the search, and the review includes studies published up to August 2024.

## 3. Results

### 3.1. Emergency and Immediate Response Situations

The Royal Spanish Academy defines an emergency as “a situation of danger or disaster that requires immediate action”. A more comprehensive definition is provided by the Spanish Civil Protection System, which describes an emergency as “a situation of collective risk caused by an event that poses imminent danger to people or property and requires rapid management by public authorities to address and mitigate the damage and attempt to prevent it from becoming a catastrophe”. These definitions highlight the critical need for rapid intervention, which is why first responders depend on audible alarms that can be activated at any time, day or night [8]. Upon hearing the alarm, first responders must immediately cease their activities, don their personal protective equipment (PPE), and be fully operational in less than 2 min for structural firefighters [10] and within 10 min for Spanish WFFs [11].

Emergency alarms have been linked to numerous adverse cardiovascular events and coronary-related deaths, particularly among United States firefighters [12,13]. These events are often attributed to the abrupt increase in heart rate (~66 bpm) following the alarm [8], compounded by subsequent strenuous physical activity [14]. Professional firefighters also report a perceived surge in adrenaline secretion during alarm responses [8], and research has shown that alarms significantly elevate blood pressure in police officers, FFs, and ambulance personnel [14]. Alarm activation also triggers an increase in salivary cortisol levels among emergency workers. Cortisol, a key hormone in the stress response, plays a crucial role in regulating metabolism, immune function, and cardiovascular dynamics while rapidly mobilizing energy to support muscular and cognitive demands [15]. However, prolonged elevations in cortisol due to chronic stress can negatively impact health, contributing to sleep disorders, digestive problems, immune suppression, and an increased risk of cardiovascular diseases [14]. In a 2016 study by Hall et al. [10], the salivary cortisol response was evaluated in 16 participants who spent four days and nights in a sleep laboratory. Participants were informed they might receive an alarm at any time during days 1, 3, or 4, with a maximum of one alarm per 24 h period. The study found significant increases in cortisol levels during nighttime alarms, whereas no significant changes were observed during daytime alarms. Over time, such nighttime disturbances could have cumulative negative effects on the health and well-being of emergency workers.

To mitigate the negative impact of emergency alarms on wildland firefighters, various strategies can be integrated into work and training programs. One effective approach is the implementation of rotating schedules that evenly distribute daytime and nighttime response duties, reducing the frequency of sleep disturbances and the cumulative cardiovascular strain associated with repeated alarm exposure [8]. Additionally, regular physical exercise has been identified as a crucial strategy for enhancing cardiovascular resilience and minimizing the adverse effects of sudden heart rate surges triggered by alarm activation, which can increase by up to 66 beats per minute [16].

### 3.2. Terrain Conditions, Equipment Weight, and Duration of Effort

The work of WFFs is characterized by three main challenges: carrying heavy loads (7–29 kg), prolonged interventions lasting over three hours, and adverse terrain conditions [2,3,4,17,18]. Each of these factors presents significant physical and physiological demands, and when combined, they considerably increase the risk of accidents. These variables are inherently dynamic, as the type of fire, the firefighter’s role, and immediate operational needs determine the specific load, duration, and terrain challenges faced.

#### 3.2.1. Carrying Heavy Loads

At an international level, the weight of the loads carried by WFFs varies depending on their location and operational requirements. In the United States, Interagency Hotshot crews carry loads averaging 28 ± 6 kg [2]. In Canada, equipment weights fluctuate based on the tools used, ranging from 4.1 kg for PPE to 28.5 kg for a water pump [19]. In Spain, Helitack crews generally carry an average load of 11.5 kg, with values ranging from 4 to 25 kg [20].

Heavy loads impose significant physiological demands, increasing metabolic cost, altering locomotion mechanics, and reducing the capacity for sustained external work. These factors result in greater energy expenditure during physically demanding tasks and predispose firefighters to a higher risk of injuries and chronic pain [5,20,21,22,23,24]. Notably, WFFs experiencing chronic pain in multiple body regions were found to carry PPE that was approximately 2.5 kg heavier than those reporting pain in a single body region [20]. Although this difference may seem small, it represents about 10% of the maximum weight carried by WFFs during deployment [20]. The impact of load weight is compounded by factors such as load dimensions, distribution on the body, and the relative load-to-body mass ratio [24]. This issue is particularly significant for female firefighters and individuals with lower body mass, for whom a typical 20 kg load may represent up to 40% of their body weight [23]. Further analysis of chronic pain and injury risk related to load carriage is presented in Section 3.10.

A study by Carballo-Leyenda et al. [17] analyzed the effects of full PPE (coveralls, gloves, helmet, and boots) on the Pack Test performance, a standardized assessment used to evaluate WFFs’ ability to perform job-related tasks. The results indicated a 12% increase in completion time when wearing full PPE (33.3 ± 3.9 min under control conditions vs. 37.2 ± 4.4 min with PPE), highlighting the substantial physiological impact associated with load carriage during firefighting operations.

PPE and transported loads create a delicate balance between operational efficiency and physiological strain, making it essential for decision-makers to carefully evaluate equipment design and work strategies. To optimize performance and minimize injury risk, ongoing research should focus on improving PPE ergonomics, reducing equipment weight, and exploring alternative load distribution methods [24]. Additionally, structured physical conditioning programs that integrate strength and endurance training have been shown to enhance load-bearing capacity and reduce injury prevalence among firefighters performing physically demanding tasks [25,26].

#### 3.2.2. Long Duration of Interventions

The duration of WFF interventions varies significantly by country. In the United States, Canada, and Australia, WFFs often work in continuous shifts that can last for consecutive days [1,27,28]. In Spain, shifts are typically 8–12 h, although emergencies may extend them to 14 h, with a minimum rest period of 10 h between shifts, as recommended by the Committee for Forest Firefighting [29].

These variations influence the intensity of effort required during interventions. Research by our group analyzed over 200 wildfires, examining the time WFFs spent in three intensity zones: low intensity (below the aerobic threshold), moderate intensity (between the aerobic and anaerobic thresholds), and high intensity (above the anaerobic threshold) [3,4]. The results indicated that as fire duration increased, time spent in moderate- and high-intensity zones decreased (Table 1), likely due to accumulated fatigue, which limits sustained high-intensity work. Moreover, existing evidence consistently shows that prolonged deployments negatively impact both sleep quantity and quality, leading to progressive fatigue accumulation and impaired alertness over time [30].

During the initial phase of firefighting, WFFs engage in intense tasks such as direct flame suppression or building fire lines, which demand high energy expenditure and physiological effort. As the fire becomes controlled, tasks shift to lighter activities, resulting in reduced intensity. Direct or mixed attack scenarios require the greatest time spent in moderate- and high-intensity zones, as proximity to flames exposes firefighters to elevated temperatures and necessitates a faster work pace to suppress fire spread, further increasing energy and physical demands (Table 1).

### 3.3. Smoke Inhalation and Its Health Effects

WFFs are exposed to significant health risks from inhalation of hazardous air pollutants released during the combustion of live and dead plant biomass (smoke) and soil dust. These exposures often occur during long shifts without respiratory protection, further increasing their vulnerability [31]. Wildfire smoke is a complex mixture of gaseous and particulate pollutants, including carbon dioxide, carbon monoxide, formaldehyde, and polycyclic aromatic hydrocarbons, among others [32].

The toxicity of the firefighting environment, particularly in wildland and wildland–urban interface (WUI) areas, has been exacerbated over the past 15 years by the intensifying climate crisis. Additionally, fires in WUI zones have led to increased exposure to hazardous fire emissions resulting from the combustion of synthetic materials. The WUI represents a hybrid environment where structural and wildland fire zones intersect, defined as “the area where structures and other human developments meet or intermingle with undeveloped wildland fuels” [33]. These changing fire dynamics pose new health challenges for firefighters, who now face not only the traditional risks associated with wildfire smoke but also exposure to additional toxic compounds generated by burning plastics, household chemicals, and other synthetic materials.

The effects of inhaled pollutants on WFFs have been predominantly studied in the short term, as highlighted in recent reviews [32,34,35,36]. These studies report notable declines in lung function and airway responsiveness following wildfire seasons compared to pre-season measurements. Moreover, significant reductions in lung function have been observed within individual work shifts, often linked to high exposure levels of levoglucosan, a specific marker of wood and vegetation combustion. Elevated levels of systemic inflammation biomarkers have also been detected in firefighters after wildfire and prescribed burn exposures, indicating heightened physiological stress.

Long-term studies are more limited and primarily rely on risk assessments and predictive models [31,37]. Booze et al. [37] conducted a health risk assessment that identified an increased likelihood of lung cancer and other non-cancer health effects in WFFs, largely attributed to prolonged exposure to benzene and formaldehyde. Similarly, Navarro et al. [31] demonstrated that particulate matter from wildfire smoke, coupled with the breathing rates typical during firefighting activities, significantly increases the risk of lung cancer and cardiovascular diseases. These risks are exacerbated by the duration of a firefighter’s career and the cumulative number of days spent managing wildfires in each fire season.

Over the last 25 years, several key strategies have been proposed to mitigate the impact of smoke exposure on WFFs. These include reducing exposure by limiting mopping-up duties in areas with high smoke concentrations whenever possible and implementing rotational assignments to evenly distribute exposure across shifts. Additionally, medical surveillance programs and the establishment of specific occupational exposure limits for WFFs have been recommended to improve long-term health outcomes [31]. Although the use of respiratory protective equipment or particulate masks remains limited, their widespread adoption is strongly encouraged to reduce exposure to airborne contaminants [36]. To ensure the effectiveness of these measures, it is crucial to consider user experience, comfort, and feasibility when selecting protective devices, promoting their seamless integration into firefighting operations. Comprehensive training on smoke hazards and proper respiratory protection practices should also be prioritized to enhance awareness and encourage compliance. Finally, operational modifications, such as relocating base camps away from high-smoke areas, should be explored to further reduce overall exposure and improve on-site safety conditions.

### 3.4. Thermal Stress and Environmental Stress in the Work Environment

The work of WFFs is characterized by high physical demands influenced by the nature of their tasks, environmental conditions, and the protective equipment they are required to wear [3]. Environmental stressors during wildfire seasons, such as temperatures ranging from 35 to 45 °C, relative humidity levels below 20%, and wind speeds exceeding 30 km·h^−1^, have been documented in Australia [38]. These factors expose WFFs to significant thermal strain, particularly when working near flames. Studies have reported average core temperatures of 38.3 ± 0.1 °C, with maximum values approaching heatstroke levels (39.2 ± 0.3 °C), compounded by impaired heat dissipation due to the use of protective equipment [3,39]. Under such conditions, excessive thermophysiological demands (marked by increases in core temperature, skin temperature, heart rate, and sweat rate) can impair physical and cognitive performance, increasing the risk of thermal stress, muscle cramps, heat syncope, heat exhaustion, or even heatstroke [21,39,40,41,42,43].

Thermal strain results from heat accumulation within the body, driven by environmental conditions, physical exertion, the use of PPE, and individual thermoregulatory capacity. It is associated with both physiological and psychological complications, including heat-related illnesses, and an increased risk of occupational accidents [38]. While PPE provides essential protection against external hazards, it also restricts heat exchange with the environment due to its high thermal resistance and low permeability to sweat vapor. This can lead to incompensable heat strain, where continuous heat storage occurs, significantly increasing the risk of heat-related injuries [44].

The most common types of heat-related illnesses include the following. (i) Heat-related muscle cramps, often an early sign of heat illnesses, caused by sodium depletion, dehydration, and neuromuscular fatigue [45]. (ii) Heat syncope, a condition characterized by fainting due to prolonged standing or high temperatures, especially during the initial phase of heat acclimatization. Risk factors include dehydration and certain medications, while acclimatization reduces susceptibility [46]. (iii) Heat exhaustion, defined by an inability to sustain physical activity in high-heat conditions, with symptoms such as excessive sweating, fatigue, and discomfort. Cognitive function typically remains unaffected [43]. (iv) Heatstroke, the most severe form of heat-related illnesses, with core temperatures exceeding 40 °C, leading to multiorgan dysfunction. This condition requires immediate treatment with rapid cooling to prevent fatal outcomes [43].

As occupational heat exposure risks increase, effective heat mitigation strategies must be implemented to maintain a safe and healthy work environment. Maintaining good physical fitness is a key factor in reducing the risk of heat-related illnesses, as a well-conditioned body adapts more efficiently to heat stress and exhibits greater thermoregulatory efficiency [43]. Individuals with high cardiovascular endurance and pulmonary capacity benefit from improved blood circulation and oxygenation, which support thermal balance during exercise and heat exposure [43,46,47].

Similarly, heat acclimatization/acclimation induces several physiological adaptations, including [48]: (i) increased sweating rate with reduced salt concentration, enhancing evaporative cooling; (ii) expanded blood plasma volume, which improves blood pressure regulation, stroke volume, and heat dissipation capacity; and (iii) improved subjective heat tolerance, resulting in lower perceived exertion and reduced thermal discomfort. These adaptations reduce the rate of body temperature rise during exercise or work in hot environments [48], which can lead to a 15% improvement in physical work capacity [49]. Acclimatization progresses rapidly, with 75–80% of adaptations occurring within 4–7 days of initial heat exposure. While aerobic fitness and heat acclimatization remain the most effective long-term strategies for managing heat stress [48], their benefits require systematic implementation over weeks or months. Additionally, cooling interventions can provide immediate relief by either [50] (i) pre-cooling strategies, which increase heat storage capacity before exercise, or (ii) per-cooling techniques, which moderate the rise in core body temperature during exercise or work in high-heat conditions. These measures can enhance work performance and safety, making them practical components of occupational heat management programs.

### 3.5. Dehydration and Its Impact on Health and Performance

Hydration during physical activity plays a critical role in maintaining physical performance and overall health, both in sports and in occupational settings, such as wildland firefighting. Any imbalance that impairs an individual’s ability to stay optimally hydrated negatively impacts both performance and well-being [51,52,53]. The American College of Sports Medicine recommends using various biological indicators to assess hydration status during physical activity, including body weight loss and urinary density measurements [54,55].

During exercise, body temperature rises, triggering thermoregulatory mechanisms such as increased blood flow to the skin (i.e., peripheral vasodilation) and sweating, which help dissipate heat [54]. The evaporation of sweat is the most effective mechanism for cooling the body, particularly in hot and dry environments [54]. However, in conditions where both temperature and humidity are high, sweat evaporation becomes impaired, leading to a progressive increase in internal body temperature [54]. This combination presents a significant thermal challenge, further exacerbating dehydration risk due to accelerated fluid loss.

Dehydration impairs performance by inducing fatigue, reducing focus, and limiting thermoregulatory efficiency. This increases the likelihood of serious health risks such as muscle cramps, heat exhaustion, heatstroke, and even death [56]. In addition, to fluid loss, sweating results in the depletion of key electrolytes, particularly sodium and chloride [57]. On average, approximately 3.2 g of salt is lost per liter of sweat, with sweat rates ranging from 1 to 1.5 L per hour of physical activity. These electrolyte losses contribute to muscle cramps, fatigue, and reduced performance capacity [54,55].

For WFFs, adequate hydration is essential to mitigate dehydration-related impairments, maintain physical and cognitive performance, and reduce the risk of accidents and injuries, which are critical in high-risk environments [18,41,58,59,60,61]. The following consequences of dehydration have been documented in WFFs [18,41,58,59,60,61]:

- Increased fatigue and muscular weakness, raising the risk of injuries and accidents during operations.
- Greater susceptibility to heatstroke, due to impaired thermoregulation when hydration levels are insufficient.
- Higher likelihood of gastrointestinal disturbances, including stomach discomfort, vomiting, diarrhea, and abdominal cramps.
- Electrolyte imbalances, potentially leading to muscle cramps, and in severe cases arrhythmias.
- Reduced cognitive function, impairing mental focus and decision-making, which can compromise safety and operational effectiveness.

Maintaining optimal hydration through regular fluid and electrolyte intake is fundamental for WFFs to perform effectively and safely during wildfire suppression activities. However, hydration alone is insufficient to meet the physiological and cognitive demands imposed by prolonged wildfire suppression efforts. Adequate rest and sleep are equally critical for recovery, decision-making, and sustained performance during extended deployments. Although hydration guidelines for athletes serve as a useful reference, their applicability to WFFs may be limited due to the use of PPE and exposure to extreme environmental conditions [62]. Nonetheless, general recommendations can still be followed. Before deployment, it is recommended to drink 350–500 mL of water within the four hours prior to activity, increasing intake if urine appears dark [54]. During deployment, it is essential to replenish fluids and electrolytes, initially consuming 400–800 mL per hour, and up to 1 liter per hour during prolonged fire suppression activities [54]. Additionally, monitoring urine color, avoiding alcohol, and controlling sodium intake are recommended strategies. Electrolyte additives have been shown to improve hydration status among WFFs, making their inclusion in rehydration protocols advisable [41]. After deployment, immediate rehydration supports recovery, and it is recommended to replace 150% of the weight lost within the first six hours following activity [57].

### 3.6. Importance of Sleep and Rest in Recovery and Performance

Sleep is a fundamental pillar of health and well-being, playing a critical role in physical development, emotional regulation, cognitive performance, and overall quality of life [63]. The recommended amount of sleep for optimal health and function varies across the lifespan, gradually decreasing from birth to adulthood, with adults typically requiring 7 to 9 h per night [63]. However, individual sleep needs can differ based on factors such as illness, prior sleep deprivation, and physiological or psychological stress [53]. Importantly, while sleep duration is a key metric, sleep quality is increasingly recognized as an essential component of overall health and performance [64]. For athletes, sleep is indispensable for optimizing performance, recovery, and quality of life [65]. Recommendations suggest athletes should aim for longer sleep durations (9–10 h per night) compared to the general population. However, evidence indicates that athletes often fail to meet these recommendations due to factors such as heavy training loads, irregular schedules, competition demands, travel, and time zone changes [63,64,65]. Insufficient sleep can impair cognitive and motor performance, reaction times, emotional stability, and mood [64].

The effects of sleep deprivation on WFFs have been extensively studied in countries such as Australia, the United States, and Canada [30,32,38,66,67,68,69]. During wildfire deployments, WFFs often sleep in temporary accommodations near the fire site or directly at the wildfire scene, using vehicles or tents [38,70]. Environmental factors such as heat, light, smoke, noise, and unfamiliar surroundings significantly compromise the quality and quantity of sleep [30,38,67,70]. As a result, WFFs report sleeping an average of 3 to 6 h per night during extended deployments lasting several days [70,71]. In the short term (2–3 days of sleep restriction), reduced sleep does not appear to significantly impair physical performance or cognitive abilities [67,70]. However, during longer deployments (14–17 days), sleep deprivation leads to notable declines in cognitive performance, increased fatigue, and greater levels of daytime sleepiness [30]. These findings underscore the importance of ensuring adequate sleep opportunities for WFFs to maintain operational performance and safety during prolonged wildfire suppression efforts.

Understanding the impact of sleep duration and quality on cognitive function, decision-making, and overall well-being is essential for developing effective mitigation strategies for WFFs. At the individual level, adopting proper sleep hygiene practices can help optimize recovery and performance [36]. Additionally, work shifts should be structured, whenever possible, to provide adequate recovery opportunities, including scheduled rest periods during shifts and sufficient sleep between shifts [69]. Furthermore, it has been suggested that quantifying the degree of impairment due to sleep restriction and establishing safe work tolerance times could help manage exertion and smoke exposure more effectively [38]. Implementing shorter exposure durations, structured task rotations, and strategic rest intervals may further enhance fatigue management and improve overall operational effectiveness [38].

### 3.7. Personal Protective Equipment and Its Influence on Health and Performance

The use of PPE is essential and mandatory for WFFs, offering critical protection against thermal exposure and other occupational risks [21,22]. Carballo-Leyenda et al. [22] analyzed thermal exposure in Spanish WFFs during wildfire interventions and found that PPE reduced heat flow by 70%, particularly in the chest and thigh areas. However, while PPE provides necessary protection, it can also reduce work capacity, impair mobility, and increase physiological strain, ultimately affecting performance and safety [21,24,72,73,74,75].

PPE, including helmets, gloves, neck protection, eyewear, and boots, significantly increases equipment weight and exacerbates thermal strain [21,22,72,76]. In one study [21], six WFFs performing a 120 min exercise under three conditions (sportswear, partial PPE, and full PPE) showed that full PPE led to nearly double the rise in core temperature compared to lighter configurations. This was attributed to restricted heat dissipation and increased metabolic heat production. Full PPE also elevated physiological variables such as VO_2max_, ventilation, and maximal heart rate compared to lighter clothing, highlighting its impact on performance [17,77].

These physiological challenges translate into reduced performance during maximal effort and field tests. For example, Carballo-Leyenda et al. [21] reported shorter exercise durations for WFFs wearing full PPE (62.4 ± 13.3 min) compared to wearing only coveralls and boots (115.5 ± 5.0 min) or sportswear (118.2 ± 20.7 min). Similarly, Phillips et al. [77] examined performance under three conditions: carrying a 20.3 kg backpack with sportswear; carrying a 20.3 kg backpack with workwear (coveralls and boots); and unloaded with sportswear. Significant reductions in effort duration were observed across these conditions, with performance while wearing workwear (793 ± 27 min) being 9% lower than when carrying the backpack with sportswear (873 ± 31 min).

PPE also impacts the Pack Test, a standardized 4.83 km course completed with a 20.3 kg load in under 45 min. WFFs’ performance significantly decreased from 41.2 ± 0.5 min in LEX to 42.6 ± 0.5 min in LW conditions. Additionally, Carballo-Leyenda et al. [20] observed a 12% reduction in Pack Test performance under full PPE (33.3 ± 3.9 min) compared to control conditions with sportswear (37.2 ± 4.4 min). These findings underscore the physiological and performance costs associated with PPE use, emphasizing the need for balancing safety and efficiency during wildfire operations.

Research on structural firefighters has demonstrated that PPE prototypes with enhanced ventilation designs and reduced layers can significantly improve physiological comfort, including reductions in core temperature, skin temperature, and overall physiological strain [78]. However, these designs are not recommended for high-heat environments or situations involving direct flame exposure, biological hazards, or chemical contaminants [78]. In the case of WFFs, further research is needed to explore PPE modifications that could help mitigate heat strain without compromising protective capabilities. Developing lighter, more breathable materials and optimizing ventilation without reducing thermal protection may provide a viable solution for enhancing both safety and performance in wildfire suppression operations.

### 3.8. Physical Load and Energy Expenditure

To quantify the total energy expenditure (TEE) of first responders, including WFFs, various methods and devices have been employed.

- Accelerometers. These devices measure the frequency and magnitude of body movements, estimating TEE based on individual characteristics such as age, sex, height, and weight. Accelerometers can operate in one plane (uniaxial) or three planes (triaxial) and have been validated under habitual living conditions using indirect calorimetry and doubly labeled water methods [79].
- Doubly Labeled Water. Considered the gold standard for estimating energy expenditure, this method involves enriching body water with isotopes of hydrogen and oxygen. The difference in the washout kinetics of these isotopes provides an accurate measure of carbon dioxide production and consequently energy expenditure. While ideal for assessing energy expenditure in real-world conditions, this technique requires a complex equipment and strict protocols [80,81].
- Metabolic Equivalents (METs). METs provide a standardized measure of energy demands, expressed as the ratio of working metabolic rate to resting metabolic rate (RMR, defined as 1.0 kcal·kg^−1^·h^−1^). For reference, 1 MET is approximately 3.5 mL O_2_·kg^−1^·min^−1^ or 1 kcal·kg^−1^·h^−1^. Physical activities are categorized based on intensity: sedentary (<1.49 METs), light (1.50–2.99 METs), moderate (3–5.99 METs), and vigorous (>6 METs) [82,83].

Using these methods, the significant energy demands faced by WFFs have been well documented internationally [2,41,58,80,84,85]. As early as 2004, the Center for Work Physiology and Exercise Metabolism (University of Montana, USA) reported a TEE for United States WFFs ranging from 2719 to 6260 kcal·d^−1^ during wildfire interventions [80]. A subsequent study 15 years later revealed similar values, with TEE ranging from 2946 to 6083 kcal·d^−1^ and an average of 4556 ± 943 kcal·d^−1^, underscoring the high physical demands of this profession [18] (Table 2).

The occupational demands of WFFs require an energy intake that matches their high caloric expenditure while maintaining consistent eating patterns during long shifts [58]. These demands vary by role and intensity throughout the fire season, typically from June to October in Spain. Sports nutrition literature emphasizes the importance of appropriate nutritional supplementation during prolonged physical activity (>2 h), which improves performance and preserves muscle glycogen [88,89].

In WFFs, adequate carbohydrate supplementation has been shown to enhance work performance during extended shifts and reduce physical and mental fatigue [90]. Marks et al. [58] found that WFFs working 14 h shifts consumed an average of 3684 ± 1493 kcal·d^−1^, resulting in an energy deficit compared to a TEE of 4556 ± 943 kcal·d^−1^. Their dietary intake was characterized by a higher-than-recommended consumption of fats and proteins, yet an insufficient carbohydrate intake, a mismatch that could negatively impact performance and long-term health. Pre- and post-fire season assessments in United States WFFs have documented increases in total cholesterol, low-density lipoproteins, and visceral fat, alongside declines in key health markers, suggesting that current dietary habits may contribute to a deterioration in metabolic health [91,92]. In general, recommended macronutrient intake for WFFs includes 6 to 10 g·kg^−1^ of body weight of carbohydrates, 1.2 to 1.7 g·kg^−1^ of protein, and 20% to 35% of total caloric intake from fats [93].

Despite their above-average fitness levels, a study of 701 Spanish WFFs [94] identified a concerning health profile, with high body fat percentages, elevated visceral fat, and unfavorable body mass index values. These were compounded by deficits in lean and bone mass, posing a heightened risk of cardiometabolic diseases [94].

### 3.9. Mental Stress and Anxiety

The increasing severity of wildfires not only results in greater environmental and economic losses but also demands substantial human and material efforts for their suppression, intensifying the impact on the personnel involved [95]. Wildfire emergencies place significant physical and mental demands on workers, often leading to heightened levels of anxiety and stress [96,97,98].

Work-related stress in WFFs is defined as the cumulative physical, environmental, and psychological burden experienced during their duties [99]. Common stressors on the fire scene include exposure to smoke, the intensity and duration of work, heat accumulation from physical exertion, challenging climatic conditions, and the unpredictable behavior of the fire itself [96,99]. Physiological responses to these stressors include increased heart rate, excessive sweating, elevated cortisol levels, and heightened momentary anxiety [96,97,99]. Under sustained or escalating stress, the resultant fatigue can impair decision-making and performance, increasing the risk of accidents, injuries, and long-term health issues [80,96,99].

These physiological responses are primarily driven by the activation of the sympathetic division of the autonomic nervous system and the hypothalamic–pituitary–adrenal axis. The sympathetic system triggers immediate reactions to perceived threats, such as increased heart rate and glucose supply to the muscles, while the hypothalamic–pituitary–adrenal axis initiates a slower response involving elevated cortisol levels [98]. In a study by Perroni et al. [100], salivary cortisol levels increased by 108.5% after a simulated firefighting intervention involving 20 professional Italian firefighters. Chronically elevated cortisol levels, as previously mentioned, can lead to adverse health outcomes, including sleep disorders, digestive issues, immune suppression, and an elevated risk of cardiovascular diseases [101].

Anxiety is defined as a state of agitation and tension characterized by both somatic and psychological responses triggered by anticipation, memory, or the experience of insecurity or threat, whether real or imagined [102]. Studies have documented significant increases in state anxiety following emergencies in both wildland and structural firefighting contexts [97,103]. For instance, a study assessed state anxiety in 24 professional WFFs at three key moments: before an emergency, immediately after the first emergency, and at the end of the 2017 wildfire season [97]. The findings revealed a 40.5% increase in state anxiety immediately following an emergency.

Fire suppression work has a profound impact on firefighters’ emotional well-being, with this effect being influenced by experience, level of responsibility, and personal circumstances, such as family and social support networks [97,98,99,100,101,102,103]. Implementing rotating shift systems to equitably distribute nighttime response duties may help mitigate the cumulative stress effects associated with frequent nighttime emergency alarms [8]. Additionally, a recent review by Drew Gonzalez et al. [104] highlights that relaxation techniques and mindfulness practices are effective strategies for reducing stress and anxiety in response to high-intensity situations. Integrating these interventions into firefighter health promotion programs could enhance both physical and psychological resilience, improving well-being and operational performance during wildfire suppression efforts.

### 3.10. Injuries and Chronic Pain Associated with the Performance

The physically demanding nature of WFFs exposes personnel to significant short-term risks, such as injuries and accidents, and long-term challenges, including chronic pain and potential disability [105]. Although studies on occupational injuries among WFFs are limited compared to structural firefighters, emerging research highlights the specific risks faced by this professional group [5,106,107,108].

Occupational injuries are defined as those caused by, contributed to, or worsened by work-related activities or exposures [109]. In WFFs, common causes of injuries include slips and falls (28%) and the use of tools and heavy load transportation (22%), with the lower extremities being the most frequently affected area (35%) [5]. For instance, Britton et al. [5] reported 1304 injuries among United States WFFs between 2003 and 2007, emphasizing the physical demands of the role. Another study by Gordon and Lariviere [107] analyzed predictors of injury in WFFs, distinguishing between less severe injuries (e.g., requiring first aid) and severe injuries (e.g., involving medical leave). Their findings revealed that 10% of participants experienced less severe injuries, while 15% suffered more severe injuries. Key risk factors included stress, age, experience, and injury history, suggesting that firefighters with greater exposure are at heightened risk. In this context, stress management interventions could play a critical role in mitigating the incidence of severe occupational injuries, helping to enhance both individual resilience and workplace safety [5,107].

Our research group further explored this issue in a sample of 217 Spanish WFFs (199 men and 18 women) [108]. A high prevalence of injuries (~76%) was observed, with age and work experience being the most influential factors. Firefighters over 35 years old and those with over 10 years of experience were significantly more likely to sustain injuries. Injuries were most common during physical training (~46%), followed by preventive work (~33%) and wildfire suppression (~20%). Tendinitis was the most frequently reported condition (~44%), followed by muscle pain (~21%) and sprains (~21%) [108]. These findings underscore the need for tailored physical training programs to reduce the high injury rates among WFFs [5,107,108].

Chronic pain is another critical issue in WFFs, defined as pain lasting three months or longer, often resulting from injuries, overuse, or cumulative trauma [110]. Chronic pain has a multifactorial origin and significantly affects quality of life and work performance [110,111]. In our study involving 221 Spanish WFFs (203 men and 18 women), a prevalence of ~60% was reported [20]. While the prevalence was similar between sexes, pain location varied, being more common in the lower extremities among women and supervisors. Age and years of service also emerged as significant factors, with firefighters over 35 years old having four times greater odds of experiencing chronic pain and those with over 10 years of service having twice the risk [20].

Given the high prevalence of overuse injuries among WFFs performing repetitive manual labor and impact-related injuries among those in leadership roles during training, it is essential to develop targeted prevention strategies that address these distinct injury mechanisms [108]. Enhancing both aerobic and musculoskeletal fitness is likely a key factor in injury prevention, as lower fitness levels have been linked to higher injury risk in physically demanding professions [25]. Furthermore, integrating safe lifting techniques and stabilization exercises into training programs could help fortify vulnerable areas, particularly the back, shoulders, and knees, which are among the most injury-prone regions. Implementing these measures could optimize the physical preparedness of WFFs, ultimately reducing injury incidence and improving operational efficiency in wildfire suppression [108].

## 4. Conclusions

The evidence underscores the critical importance of maintaining high levels of physical fitness in WFFs to safely and effectively meet the unique demands of their profession. This review highlights the need for targeted strategies to address key challenges, including mitigating thermal stress, ensuring adequate hydration, optimizing recovery through sleep and rest, and enhancing respiratory protection. Additionally, integrating specific physical and psychological assessments into selection and training programs could further improve readiness and reduce occupational risks.

Future research should prioritize interventions that balance immediate performance with long-term health outcomes, such as personalized fitness programs, improved nutrition plans, and accessible mental health support. These measures are essential not only for enhancing operational efficiency but also for safeguarding the well-being of WFFs throughout their careers. 

## Figures and Tables

**Table 1:** Intensity analysis during wildfire suppression (Rodríguez-Marroyo et al. [3,4]).

Wildfire Type	Percentage of Maximal Heart Rate (%)	Time at Low Intensity (%)	Time at Moderate Intensity (%)	Time at High Intensity (%)
By type of work				
Direct attack	67.2 ± 1.1	66.1 + 2.5	27.0 + 1.5	6.9 ±1.0
Indirect attack	58.1 ± 1.6	84.4 ± 2.6	12.9 ± 2.1	2.7 ± 0.6
Mixed attack	66.3 ± 0.9	64.0 + 2.1	28.8 + 1.6	7.1 ± 0.8
By duration				
<1 h	70.8 ± 0.8	57.1 ± 2.4	35.0 ± 2.1	7.9 ± 1.2
1–3 h	67.0 ± 0.4	62.8 ± 2.0	30.2 ± 1.7	7.0 ± 0.9
>3 h	62.0 ± 1.1	76.5 ± 2.1	20.5 ± 1.5	3.0 ± 0.6

Note: The table summarizes heart rate data collected during wildfire suppression. Intensity zones are defined as follows: low intensity (below the aerobic threshold), moderate intensity (between the aerobic and anaerobic thresholds), and high intensity (above the anaerobic threshold).

**Table 2:** Energy expenditure studies in wildland firefighters.

Study	Sample	Measurement Method	Measurement Period	Energy Demand
Ruby et al. [80]	17	Total energy expenditure (TEE) estimated using doubly labeled water	5 days of real wildfire suppression	4878 ± 716 kcal (male), 3541 ± 718 kcal (female)
Heil [86]	10	Energy expenditure measured using an electronic activity monitor (uniaxial accelerometer) to estimate TEE (total·d^−1^) and activity energy expenditure (AEE) during suppression	21-day wildfire suppression shift	4768 ± 478 kcal (TEE, per workday), 2585 ± 406 kcal (AEE, during wildfire suppression)
Cuddy et al. [18]	15	Estimation of TEE using a triaxial accelerometer	3-day wildfire suppression shift, with an average of 11.4 ± 0.7 h of work·d^−1^	4556 ± 943 kcal (TEE, per workday), 2381 ± 746 kcal (AEE, during wildfire suppression), 10 kcal·min^−1^ of work
Robertson et al. [87]	21	Energy expenditure over 24 h estimated using MET values from a compendium of physical activities	2014 wildfire season (June to September)—divided into three phases: base work (training and preventative tasks), initial attack (direct attack), and extended attack (indirect attack, prescribed burns, mop-up, and cleaning)	Base work: 2842 ± 650 kcal; initial attack: 4538 ± 1006 kcal; extended attack: 4029 ± 1165 kcal

Note: The table summarizes the energy expenditure of wildland firefighters under different conditions and using various measurement methods. The data highlight the high caloric demands of firefighting, ranging from approximately 2700 kcal·d^−1^ to over 6000 kcal·d^−1^, depending on task intensity and duration. The variation in methods (e.g., doubly labeled water, accelerometers) underscores the diverse approaches to quantifying the physiological strain associated with wildfire suppression.
